# The Surgical Treatment of Stone

**Published:** 1912-02-03

**Authors:** John Ward Cousins

**Affiliations:** Consulting Surgeon to the Royal Portsmouth Hospital.


### THE SURGICAL TREATMENT OF STONE.

### A Review of its Practice during the Last Sixty Years.—III.*

By JOHN WARD COUSINS, M.D., F.R.C.S., Consulting Surgeon to the Royal Portsmouth Hospital.

### Spontaneous Discharge of Stones.

The spontaneous discharge of small and elongated calculi occasionally occurs in young male children. Sometimes they get impacted in the upper part of the urethra, and then I have had to push them back again into the bladder and remove them afterwards by operation. At other times they have got as far as the membranous portion, and in this position they have been easily removed by incision. Many years ago a doctor in the country asked me to see a little boy with him suffering from the symptoms of vesical calculus, and on examination the diagnosis was at once confirmed and it was arranged that I should do the operation; but, at the last moment, the anxious parents decided to take their child to Mr. Thomas Bryant, the distinguished surgeon of Guy's Hospital, and they speedily returned with a letter to their medical attendant which contained a kind reference to myself. The day was fixed again; but fortunately my surgical enthusiasm was doomed to receive another check, for just as I was leaving my residence to perform the operation the delighted father handed me the stone which his little son had safely passed during the night.

### Character and Composition of Calculi.

The calculi which I have removed during my practice include many specimens which vary much in size, shape, and composition. The small stones were for the most part uric acid concretions, and many of the larger were compounds of urates combined with phosphatic layers, caused by prolonged vesical irritation and the alkaline condition of the urine. Four of the specimens were oxalate of lime calculi, very irregular in shape and covered with hard nodules. One of these I removed from a child four years old, who had suffered a long time with acute pain and constant bladder irritability. It was a very interesting concretion, and was made up of sharp spikes projecting from a very small central formation. Two of these projecting processes were broken during the operation. One small calculus presented several smooth marks on its surface, and on searching the bladder carefully again I found another stone. Two calculi taken from an adult patient exhibited very unusual facets, and they completely fitted into each other, forming a perfect ball and socket joint. I have met with only one fixed stone, and this was partly encapsuled near the base of the bladder. Fortunately it was extracted without fracture after several efforts with curved forceps. The embedded portion was thick and rough, and about half an inch in length. It could have been much more easily removed by the supra-pubic operation. Two of my specimens had undergone fracture and molecular disintegration. One was taken from a patient suffering from vesical ulceration and prostatic disease; the other occurred in an emaciated young man of twenty-eight years of age, who had been bedridden for a long period from obstructed micturition, occasional hæmorrhage and cystitis. The lateral operation was performed, and four ounces of soft and fœtid concretion were removed, and the walls of the bladder were found coated with phosphatic deposit. After prolonged antiseptic treatment he appeared to make good recovery, but soon he returned to me suffering from a relapse of his old trouble, and three months after my first operation I opened his bladder again and took away several fragments of hard calculus, and this was followed by permanent relief. I sent the concretion to my old friend the late Dr. William Ord, who was then investigating the spontaneous changes in calculi. He only returned a part of the specimen to me, and at his request I gave him the remainder, which I believe is now to be seen in the museum of St. Thomas' Hospital.

### Diagnosis of Stone in the Bladder.

There are many symptoms, such as vesical irritation, occasional hæmorrhage, and straining at micturition, which indicate when persistent the probability of stone, but no positive evidence can be obtained without an exploration of the bladder. Formerly the operation of sounding was looked upon as a very trifling proceeding, and it was undertaken without any preparation of the patient or any surgical precautions. I have known alarming symptoms to follow the introduction of an instrument even when not attended with special difficulty.

Now it has been my plan for many years in every case of suspected calculus to prepare the patient by at least twenty-four hours' complete rest, and also to secure a report on the condition of the urine before making any examination of the bladder.

As a general rule an anæsthetic has been employed in all my examinations, and before putting in the instrument the urine has been removed and a few ounces of warm sterilised water injected. The sound I have always used has a short beak somewhat bulbous at the point, and a long handle just large enough to fit loosely the urethra. It is easily introduced by a gentle gliding movement, and every part of the cavity can be rapidly explored. With the handle slightly depressed the beak passes over the fundus, turned first to one side and then the other, and after slightly withdrawing the handle and imparting to it a rotatory movement, the beak is made to explore the prostatic region. Sometimes these manipulations carefully repeated several times have proved unsuccessful, but when this is the result the pelvis should be raised on a sand-bag so that the concretion may be moved into a more favourable position, and detected by touching it with the sound as well as by an audible ring. Now when these impressions on the hand and ear were too feeble to be regarded as positive evidence, it became necessary to repeat the sounding after a delay. The stethoscope placed over the pubic region has sometimes proved useful, and has appeared to render

* Previous articles in this series appeared in The Hospital of Jan. 20 and Jan. 27.

sounds more audible. I have never used the lithotrite for the purpose of estimating the size of a calculus before performing a lithotomy operation, but have rather trusted to the indications obtained by the sound respecting the shape, roughness, and irregularity of the surface. Like many other surgeons I relied upon these long-recognised methods, and have had sometimes to confess that the evidence obtained by them was not conclusive, and surgical interference quite out of the question until positive proof of a concretion was obtained.

### Röntgen Photography.

Now I have touched upon these practical details for the purpose of exhibiting how our means for accurate diagnosis have been assisted during the last twenty years by the developments of collateral science. As soon as the results of the Röntgen photography were made known the recognition of foreign bodies in the internal organs was soon found to be possible, and this proved to be the beginning of the great progress which has been made in the detection of vesical and renal calculi. The $x$-ray work has been of immense service in many cases of difficulty, and has not only clearly brought forth the presence and shape of stones which had before escaped the touch of the sound, but has also indicated their position whether in the bladder or lodged somewhere outside in the ureter or kidney. In addition to this the character of the shadows actually suggests the nature of the formations, for faint pictures are the outcome of calculi composed of uric acid and its compounds, and that the depth and definition of the shadows have always a direct relation to the amount of lime salts present in the concretions.

Again, the rapid advance of electro-medical science has led to the development of modern methods of illuminating the cavity of the bladder. The first instrument of the kind was the invention of Prof. Nitze, of Vienna, but has been much improved by the incandescent lamp.

### The Cystoscope.

The cystoscope has proved of great assistance in the diagnosis and treatment of vesical disorders, and with it the interior of the bladder can be lighted up and a complete picture obtained of the vessels and surface of the lining membrane, and a calculus also may be brought to light which had been long hidden in a thick coating of mucus or some other inflammatory deposit. By its help stones have been discovered under the prostate or encysted in saccules of the vesical wall, and others have been clearly seen impacted in the orifice of a ureter, and then the exact definition of their location has greatly assisted in their removal.

Before the introduction of electric illumination the diagnosis of many disorders was certainly impossible without an exploratory operation, but this is not the case to-day. By the aid of the modern cystoscope the existence of many structural changes, papillomata and malignant growths can be detected. The instrument is now made with special arrangements for irrigation and evacuation of the bladder, and also for the introduction of fine catheters adapted for ureteral exploration. For many of these valuable improvements we are indebted to Mr. E. Hurry Fenwick.

In concluding my review of past events connected with the treatment of vesical stone, I venture to express the hope that it may prove interesting to many students and practitioners just beginning their professional career.

It is a remarkable fact that by the recent progress of surgical science the old and historic lateral operation of Cheselden is rapidly passing into the shade, and that the still older and long discarded supra-pubic method is actually in the ascendant throughout the country. But this revolution is not difficult to explain, as the re-introduction of this ancient procedure is now surrounded by all the improvements and precautions of modern practice. It is far more easy to reach the bladder and extract the stone through the abdominal wall than by following the perineal route, and the performance requires much less dexterity, as the eye in every stage of the operation is of great assistance.

During the last fifteen years the preference for the high method has been steadily progressing, and the result of the inquiry carried out by Mr. William Haslane * and mentioned in his valuable Lettsonian lectures did not excite any surprise among the surgical profession. He states the result of his own experience in these words: "It is now, with hardly any exception, the only cutting operation resorted to."

With reference to litholapaxy, it undoubtedly holds second place in British practice, but it is not making very rapid progress at present. There are, however, some surgeons who have had special opportunities of practising stone crushing, and in this way they have been enabled to obtain both skill and reputation in its performance. Litholapaxy is not yet perfect in every detail, but I am confident that it will soon be more generally utilised.

It appears scarcely necessary to add that the surgeon in every case still has all the responsibility of selecting the form of surgical interference, and he will, of course, consider the age and general condition of his patient, and will get a full report on the condition of the urine, and estimate the size and hardness of the calculus, and also the state of the bladder and the whole urinary track.

The following statement fairly expresses the present tendency of the surgical practice for stone in the bladder in our country:—

Men.—Litholapaxy for stones of moderate size and hardness. Supra-pubic lithotomy for all large calculi and cases complicated with enlarged prostate; chronic cystitis, and septic disturbance in any part of the urinary track.

Boys.—All small stones, litholapaxy. All other concretions supra-pubic lithotomy.

Women and Young Girls.—Dilatation and extraction through the urethra for all small stones. Larger calculi, crushing with lithotrite and irrigation, and extraction through an evacuator, or supra-pubic lithotomy.

*British Medical Journal*, March 11, 1911.

